# Ferroptosis in Vascular Diseases: A Mechanistic and Immunological Perspective on Therapeutic Targeting

**DOI:** 10.3390/antiox15040502

**Published:** 2026-04-17

**Authors:** Yiyang Cui, Pengyan Zhu, Meixiu Jiang

**Affiliations:** 1 The Queen Mary School, Jiangxi Medical College, Nanchang University, 999 Xuefu Road, Nanchang 330031, China; 2 Jiangxi Province Key Laboratory of Bioengineering Drugs, The National Engineering Research Center for Bioengineering Drugs and the Technologies, Institute of Translational Medicine, Jiangxi Medical College, Nanchang University, 999 Xuefu Road, Nanchang 330031, China

**Keywords:** ferroptosis, cell death, vascular diseases, mechanisms, inflammation

## Abstract

Vascular diseases impose a heavy global burden, yet existing therapies have limitations, necessitating novel drug targets. Ferroptosis, an iron-dependent, lipid peroxidation-driven form of cell death, acts not only as an initiator of metabolic collapse but also as a sterile inflammatory trigger by releasing damage-associated molecular patterns (DAMPs) and activating pro-inflammatory pathways. In this paper, we propose the “ferroptosis–inflammation circuit” as a self-amplifying loop where ferroptosis fuels inflammation and the inflammatory microenvironment reciprocally promotes ferroptosis via cell type-specific mechanisms. Although ferroptosis in cardiovascular diseases has been reviewed, its immunopathological role in specific vascular diseases and how macrophages, neutrophils, T cells, and vascular cells collaboratively drive pathology through this circuit remains underexplored. The unique perspective of this review is a systematic focus on the dynamic interplay between ferroptosis and immune responses within the vascular wall, moving beyond static metabolic descriptions. We synthesize evidence linking ferroptosis to atherosclerosis, pulmonary hypertension, stroke, aneurysms, and aortic dissection, emphasizing its immunological dimension across cell types. By defining the ferroptosis–inflammation circuit and its cell type-specific patterns, we reposition ferroptosis as a core pathological hub that couples metabolic dysregulation, immune activation, and vascular remodeling. Understanding this circuit may open novel therapeutic avenues for targeting the ferroptosis–immune interface.

## 1. Introduction

Vascular diseases are a group of disorders that affect blood vessels in the circulatory system, including cardiovascular and cerebrovascular diseases, etc. [1]. The incidences of morbidity and mortality related to vascular diseases continue to increase annually, imposing a healthcare burden on society worldwide. The risk factors that cause the increased incidence of vascular diseases include high blood pressure, unhealthy dietary habits, a high fasting plasma glucose level, smoking, an increased urbanization rate, long-term sedentarism, and sex. Thus, the field investigating the prevention, treatment, and prognosis of vascular diseases has been a crucial focal point in medical research in recent years [2]. Despite decades of effort, the number of individuals who suffer from vascular diseases remains very high and traditional drug treatment may have a low therapeutic index, rapid clearance rate, or systemic side effects [1,3]. Therapies for vascular diseases, such as statins and antithrombotic agents, face challenges related to systemic exposure and drug–drug interactions [4]. Conventional treatments, including angioplasty, atherectomy, and bypass surgery, are invasive and may be ineffective for local macrovascular diseases [5]. The modest and inconsistent benefits observed in recent phase II/III clinical trials of angiogenic gene therapy [6], coupled with persistent safety and efficacy concerns regarding nanoscale therapeutic strategies [5], underscore the urgent need to identify and validate novel therapeutic targets for vascular diseases.

Since Dixon first proposed ferroptosis in 2012 and identified its characteristics as intracellular iron accumulation, increased lipid peroxidation, mitochondrial alterations, the accumulation of free radicals and depletion of glutathione, ferroptosis has become a hotspot [7]. Ferroptosis, a type of cell death, is caused by the extreme accumulation of lipid-based reactive oxygen species mediated by iron. The roles of ferroptosis in neurological and neoplastic diseases have been extensively studied in recent years. Svobodová H found that the accumulation of free iron and ferritin was associated with amyloid plaque formation in the cerebral cortex to promote Alzheimer’s disease [8]. Ferroptosis was identified as an important mechanism of the pathogenesis of Parkinson’s disease [9]. Currently, researchers have found that ferroptosis plays an important role in the occurrence and progression of vascular diseases, which indicates that ferroptosis may be a promising target for the treatment of vascular diseases [10].

**Figure 1:**
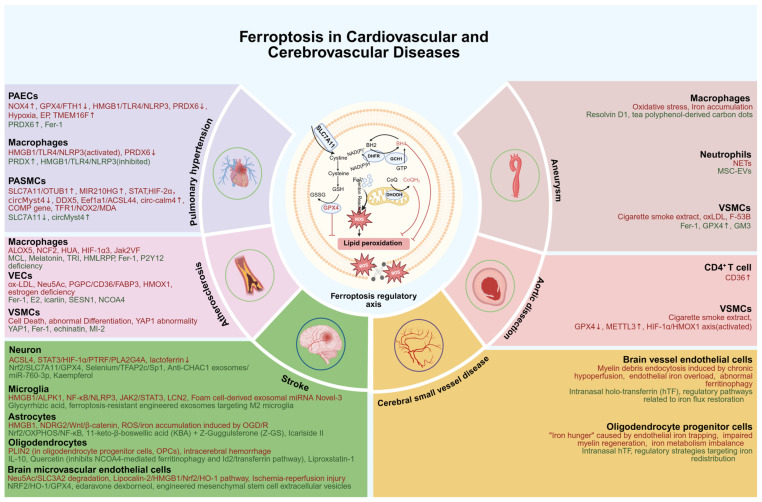
Emerging roles of ferroptosis in cardiovascular and cerebrovascular diseases. Ferroptosis, an iron-dependent form of regulated cell death, plays critical roles in the pathogenesis of diverse cardiovascular and cerebrovascular disorders. The central diagram illustrates the core molecular machinery of ferroptosis, including iron metabolism, glutathione peroxidase 4 (GPX4) defense system, and lipid peroxidation pathways. Surrounding panels depict the cell type-specific mechanisms and regulatory factors involved in atherosclerosis, aneurysm, aortic dissection, cerebral small vessel disease, stroke, and pulmonary hypertension, highlighting the potential of ferroptosis as therapeutic strategy for these diseases (↑: rise, ↓: drop; color; Red: promotion of ferroptosis, Green: inhibition of ferroptosis). Created in BioRender. Pengyan, Z. (2026) https://BioRender.com/nw05m8v.

In this review, we performed a systematic literature search in the PubMed database to identify relevant studies published up to March 2026. The search query included key terms such as “ferroptosis”, “vascular disease”, “atherosclerosis”, “stroke”, “aortic aneurysm”, “pulmonary hypertension”, “endothelial dysfunction”, and “mitochondrial dysfunction”, with appropriate Boolean operators. Only peer-reviewed original research and review articles written in English were included. Studies focusing on non-vascular cell types or unrelated pathological processes were excluded. Through this process, we summarize recent advances in the roles of ferroptosis in vascular diseases, with a focus on the interplay between ferroptosis and inflammatory responses. We highlight the self-reinforcing vicious cycle formed by these two processes, which is supported by emerging experimental evidence rather than theoretical speculation. We also discuss the contributions of ferroptosis in different vascular cell types and highlight the therapeutic potential of strategies targeting ferroptosis for treating major cardiovascular and cerebrovascular diseases. We also highlight the potential of targeting ferroptosis for the treatment of these conditions. The core molecular mechanisms of ferroptosis and its cell type-specific regulatory patterns in major vascular diseases are systematically depicted in the following schematic diagram (Figure 1).

## 2. Mechanisms of Ferroptosis: From Metabolic Priming to Bilateral Circuit Execution

Ferroptosis is a form of programmed cell death, the occurrence of which is critically dependent on iron and driven by lipid peroxidation processes. Beyond being a terminal form of metabolic collapse, it is increasingly recognized as a hierarchical failure of cellular homeostasis where mitochondrial bioenergetic failure and oxidative stress intersect with vascular aging [11]. This process follows a precise trajectory from upstream metabolic priming to the intermediate collapse of core biochemical axes, ultimately resulting in a bilateral “ferroptosis–inflammation circuit”.

### 2.1. Upstream Priming: Nutrient Sensing and Metabolic Dysregulation

The initiation of ferroptosis in the vasculature is increasingly linked to the systemic disruption of nutrient-sensing pathways, which govern the energetic landscape of endothelial and smooth muscle cells [11]. Decreased sirtuin-1 (SIRT1) activity and reduced AMP-activated protein kinase (AMPK) activation in aged vessels lead to a “pro-ferroptotic state” by disrupting the metabolic flexibility required to maintain cellular antioxidant shields [11,12]. Cells take up iron via the transferrin receptor 1 (TfR1), and its excess is stored in ferritin. Importantly, age-related iron overload, which is often exacerbated by the downregulation of the transmembrane receptor Robo4, serves as an endogenous upstream trigger that promotes cerebral endothelial senescence and sensitizes cells to subsequent oxidative insults [13]. Muckenthaler et al. demonstrated that dysregulated iron homeostasis acts as a pro-inflammatory stimulus, activating the TLR4/NF-κB pathway in vascular cells [14]. Furthermore, the iron exporter Ferroportin (FPN) is essential for maintaining cellular iron efflux, as its downregulation leads to iron overload, which triggers the release of damage-associated molecular patterns (DAMPs) from dying vascular cells [15,16]. Metabolic interventions, notably time-restricted feeding (TRF), have been shown to rejuvenate the mitochondrial transcriptome and activate adaptive networks to restore vascular respiration, thereby interrupting this upstream priming phase [11,12].

### 2.2. Intermediate Hub: Core Execution Machinery and Mitochondrial Sabotage

The essential execution mechanism of ferroptosis focuses on the catastrophic collapse of primary biochemical axes, a process that integrates traditional lipid metabolism with intermediate mitochondrial sabotage [17,18]. Characterized by the buildup of lipid hydroperoxides on polyunsaturated fatty acids (PUFAs), ferroptosis relies on the activities of acyl-CoA synthetase long-chain family member 4 (ACSL4) and lysophosphatidylcholine acyltransferase 3 (LPCAT3) to incorporate PUFAs—particularly phosphatidylethanolamines containing arachidonic acid (AA) or adrenic acid (AdA) [17,19]—into membrane phospholipids (PUFA-PLs). This classic execution is significantly amplified within the mitochondria, where Complex I-derived ROS act as key initiators of lipid peroxidation [20]. This redox sabotage is further intensified by bioenergetic failure—a central feature of metabolic vascular disease—which prevents the ATP-dependent maintenance of core defense systems, effectively lowering the threshold for cell death [11].

Mechanistically, these PUFA-PLs are oxidized by lipoxygenases (ALOXs) or through non-enzymatic Fenton chemistry to generate lipid peroxides (PLOOH). Crucially, the mitochondria undergo significant lipid remodeling during this phase. The accumulation of lipid hydroperoxides, especially cardiolipin, on mitochondrial membranes is considered an essential terminal step that links organelle damage to the global collapse of the cell [18,20]. This mitochondrion-associated ferroptosis is further regulated by MFN2-dependent dynamics, where MFN2 overexpression can suppress the mitochondrial translocation of ACSL4 to protect the microcirculation [21].

### 2.3. Antioxidant Defense Systems: Metabolic and Mitochondrial Checkpoints Against Ferroptosis

The cell possesses several defense axes to neutralize lipid peroxides, primarily the SLC7A11/GSH/GPX4 axis.

The GPX4-GSH axis is a classic antioxidant pathway. Glutathione peroxidase 4 (GPX4) reduces lipid hydroperoxides in a glutathione (GSH)-dependent manner, with its activity regulated by GSH levels and selenium availability. It prevents ferroptosis by converting lipid hydroperoxides into non-toxic lipid alcohols [22]. Stockwell et al. identified that the inhibition of the System Xc− (SLC7A11/SLC3A2) results in GSH depletion, rendering vascular cells vulnerable to ferroptosis-driven inflammation [23].

Independent of GPX4, ferroptosis suppressor protein 1 (FSP1) exhibits antioxidant activity by reducing ubiquinone (CoQ10) to ubiquinol (CoQ10H_2_), acting as a lipid-soluble antioxidant to neutralize lipid free radicals [22]. The molecular mechanism by which FSP1 regulates ferroptosis relies on its control of ubiquinone metabolism. This defense is complemented by mitochondrion-specific systems; for instance, dihydroorotate dehydrogenase (DHODH) operates alongside mitochondrial GPX4 to inhibit ferroptosis in the mitochondrial inner membrane [17,19]. This protein converts oxidized ubiquinone to reduced ubiquinol utilizing NAD(P)H. Ubiquinol, acting as a lipid-soluble antioxidant, effectively scavenges lipid peroxy radicals that drive ferroptosis. Research has shown that specifically inhibiting FSP1 activity significantly enhances the effects of GPX4 inhibitors, a synergistic mechanism that has been validated in several malignant tumor models [24]. Notably, the efficacy of these antioxidant shields is determined by mitochondrial bioenergetics, as ATP is required for GSH synthesis [11].

The GCH1-BH4-phospholipid metabolism axis functions as a further regulatory hub within the cellular ferroptosis defense system, driving the endogenous biosynthesis of the antioxidant mediator BH4, modulating coenzyme Q10 metabolic levels, and specifically inhibiting the peroxidation of atypical phospholipids bearing bis-polyunsaturated fatty acyl tails [25]. From a molecular mechanistic perspective, BH4, a potent free radical scavenging antioxidant, maintains membrane lipid peroxidation homeostasis by neutralizing lipid free radicals. Its antioxidant activity relies on the dihydrofolate reductase (DHFR)-mediated BH4 regeneration cycle. By specifically inhibiting DHFR, methotrexate leads to BH4 depletion, an effect that synergizes with GPX4 functional inhibition to produce synthetic lethality [26]. Notably, lipid-soluble antioxidants such as vitamins K/E possess similar ferroptosis-inhibiting functions [27], revealing a networked regulation of multi-level antioxidant defense systems within the cell.

### 2.4. A Conceptual Framework: The Bilateral Ferroptosis–Inflammation Circuit

While the link between cell death and immune responses has been widely recognized, we propose a formalized conceptual framework—the “bilateral ferroptosis–inflammation circuit”—to uniquely define the self-propagating synergy between metabolic failure and sterile inflammation across diverse vascular pathologies. This bilateral model posits that ferroptosis does not function merely as a terminal metabolic event but as a self-propagating driver through two interconnected dimensions.

The first dimension involves the cell-specific execution of metabolic death, where initial triggers, such as iron overload and nutrient-sensing dysregulation, ignite lipid peroxidation, leading to the terminal rupture of the plasma membrane. Consequentially, these ferroptotic vascular cells release immunogenic DAMPs, particularly high mobility group box 1 (HMGB1), which serve as the primary biochemical bridge to the second dimension. The second dimension is the immunogenic propagation phase, where these DAMPs activate the TLR4/NF-κB and NOD-like receptor family pyrin domain containing 3 (NLRP3) inflammasome pathways in neighboring innate immune cells, such as macrophages and neutrophils [17,28].

**Figure 2:**
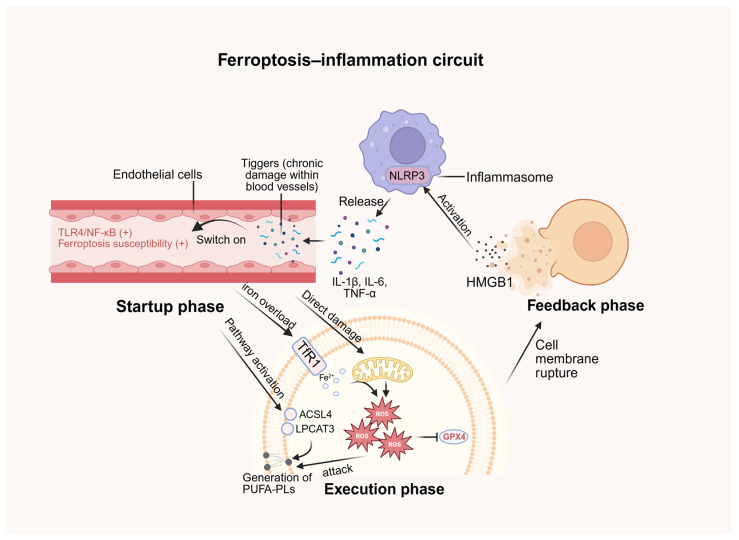
Schematic illustration of the proposed bilateral ferroptosis–inflammation circuit in vascular disorders. Chronic vascular damage initiates the startup phase by inducing TLR4/NF-κB activation and ferroptosis susceptibility in endothelial cells. In the execution phase, iron overload, mitochondrial ROS accumulation and ACSL4/LPCAT3-mediated PUFA-phospholipid peroxidation overwhelm GPX4-dependent antioxidant defenses to trigger ferroptotic cell death. Finally, in the feedback phase, ruptured ferroptotic cells release HMGB1, which activates the NLRP3 inflammasome in immune cells to induce the secretion of pro-inflammatory cytokines (IL-1β, IL-6, and TNF-α), which in turn further promote endothelial ferroptosis, forming a self-amplifying vicious cycle that exacerbates vascular injury. Abbreviation: TLR4, Toll-Like Receptor 4; NF-κB, Nuclear Factor Kappa-light-chain-enhancer of Activated B Cells; NLRP3, NLR Family Pyrin Domain Containing 3; IL-1β, Interleukin-1β; IL-6, Interleukin-6; TNF-α, Tumor Necrosis Factor-alpha; HMGB1, High-Mobility Group Box 1; TfR1, Transferrin Receptor 1; Fe^2+^, Ferrous Ion; ACSL4, Acyl-CoA Synthetase Long-Chain Family Member 4; LPCAT3, Lysophosphatidylcholine Acyltransferase 3; PUFA-PLs, Polyunsaturated Fatty Acid-containing Phospholipids; ROS, Reactive Oxygen Species; GPX4, Glutathione Peroxidase 4. Created in BioRender. Pengyan, Z. (2026) https://BioRender.com/i61c7h5.

Crucially, the bilateral nature of this circuit is established when these activated immune cells secrete pro-inflammatory cytokines (e.g., TNF-α and IL-6), which feedback to surrounding vascular cells to further suppress the SLC7A11/GPX4 axis and stimulate mitochondrial ROS production [17]. This self-amplifying feedback loop ensures that localized metabolic injury is amplified into systemic vascular inflammation. By establishing this integrative model, we propose that the “ferroptosis–inflammation circuit” represents a critical pathological axis that contributes to the chronic progression of atherosclerosis, aneurysms, and neurovascular degeneration. Rather than acting as an isolated event, this circuit is hypothesized to function as a self-propagating amplifier of the initial vascular injury. This perspective suggests that future therapeutic strategies may benefit from simultaneously targeting both the metabolic “engine” of ferroptotic death and the inflammatory “amplification” of the circuit to achieve sustained vascular protection. A schematic diagram is shown in Figure 2.

## 3. Ferroptosis in Cardiovascular Diseases

### 3.1. The Role of Ferroptosis in Atherosclerosis

Atherosclerosis (AS) is characterized by the accumulation of fatty and fibrous material in the arterial intima with progressive calcium deposition. AS involves the dysfunction of endothelial cells, macrophages, and vascular smooth muscle cells and is a leading cause of death in the modern society, imposing a heavy social burden on both developed and developing countries [29]. While lipid-lowering therapies, which typically include statins and PCSK9 inhibitors, have demonstrated effectiveness against atherosclerosis, the risk of cardiovascular events remains significant in the general population [30]. As a result, non-lipid-lowering therapies present a new insight into the treatment of AS [31]. With the development of sophisticated technologies, many new factors or mediators have been proven to contribute to the development of atherosclerosis. One of the new targets is ferroptosis because some same enzymes, transcription factors, and signaling pathways are involved in both ferroptosis and the occurrence of AS [32]. Recently, many researchers have studied the relation between the mechanism of ferroptosis in different cells and the development of AS to find new therapeutic targets.

#### 3.1.1. Macrophages

Macrophage death is a mixed blessing in the development of AS. Moderate macrophage death has positive effects in the initial stage of AS by decreasing the release of metalloproteinases and easing the inflammatory response, while uncontrolled macrophage death and ineffective efferocytosis during advanced atherosclerosis exacerbate inflammatory responses, promote the formation of a necrotic core enriched in lipids, and destroy the stability of atherosclerotic plaques [33,34].

The ferroptotic cascade in these cells is initiated by diverse molecular triggers that predominantly converge on the disruption of antioxidant defenses, making cells susceptible to lipid peroxidation. Li M et al. identified an initiation step driven by oxidative stress resulting from ROS generation mediated by the upregulation of 5-lipoxygenase (ALOX5) and P67phox (NCF2) during macrophage polarization, thereby causing ferroptosis and exacerbating AS. However, the mechanism by which ALOX5 mediates ferroptosis in atherosclerotic plaques requires further study [35]. Liu et al. showed that Jak2VF expression in the erythroid lineage caused RBCs to accumulate more ROS and lipid hydroperoxides that were subsequently delivered to macrophages through erythrophagocytosis, leading to increased macrophage ferroptosis in atherosclerotic plaques and an increased number of necrotic cores, thereby exemplifying initiation driven by oxidative stress resulting from the ROS burden [36]. Furthermore, a metabolic initiation step exists. Yu et al. demonstrated that a high uric acid level suppresses NRF2/SLC7A11/GPX4 signaling in macrophage-derived foam cells to increase their ferroptosis and thereby promote AS [37]. Hu et al. showed that P2Y12 receptor deficiency reduced NF-κB p65 phosphorylation and then inhibited hepcidin expression in macrophages, thereby preventing FPN1 degradation as a protective mechanism against ferroptosis and AS [38,39]. These oxidative, metabolic and receptor-modulated triggers represent endogenous disease mechanisms. The molecular pathways regulating ferroptosis execution have been elucidated through studies using pharmacological interventions, as detailed below.

The essential mechanism of ferroptosis execution in macrophages depends on three principal axes. First, the activation of the amino acid antioxidant defense axis (such as SLC7A11/GSH/GPX4) inhibits ferroptosis, preventing AS development. Luo et al. demonstrated that micheliolide (MCL) competitively bound KEAP1 Arg483, releasing NRF2 from the KEAP1/NRF2 complex to increase glutathione peroxidase 4 (GPX4) and Xap5 circadian timekeeper (xCT) expression, thereby improving mitochondrial function, reducing oxidative stress, inhibiting lipid peroxidation, and finally suppressing ferroptosis [40]. Lin et al. showed tricetin activates the same NRF2/GPX4 and NRF2/xCT pathways to reduce ferroptosis in macrophages [41]. Moreover, Tao et al. revealed that melatonin also can reduce macrophage ferroptosis by activating the NRF2/SLC7A11/GPX4 signaling pathway [42].

Second, the inhibition of the lipid peroxidation axis attenuates ferroptosis. Shi et al. [43] demonstrated that Maijitong granule (MJT) decreased the expression of acyl-CoA synthetase long-chain family member 4 (ACSL4) and lysophosphatidylcholine acyltransferase 3 (LPCAT3) to inhibit the synthesis of lipid peroxidation substrates, preventing AS [43]. Shi et al. also found that MJT decreased DMT1 expression via STAT6 to inhibit DMT1-mediated iron uptake and increased ferritin heavy chain 1 (FTH1) expression to bind free ferrous iron in macrophages, showing that MJT also reduced ferroptosis via the inhibition of the third core mechanism of ferroptosis, iron metabolic disorder [43]. Interestingly, MJT also activated the SLC7A11/GSH pathway to promote lipid peroxide clearance in atherosclerotic plaques and macrophages [43]. The multi-axis effects were also observed with additional modulators. Zang et al. found that 2-acetamidophenol reduced cellular Fe^2+^, MDA, and ROS levels, increased GPX4 expression, and increased the expression of genes associated with GSH synthesis and ferrous ion transport in macrophages [44]. Yang et al. showed that HMLRPP NPs can increase the GSH level in macrophages and restore Nrf2, SLC7A11, GPX4, transferrin receptor (TfR), and ferroptosis-suppressor-protein 1 (FSP-1 protein) levels in macrophages in AS lesions, preventing disease development [45]. In a word, effective ferroptosis modulation in macrophages requires the engagement of principal regulatory axes, whether through single-axis restoration or multi-axis coordination, providing the mechanistic foundation for ferroptosis in atherosclerotic plaque progression and offering multi-target therapeutic strategies.

#### 3.1.2. Vascular Endothelial Cells

The dysfunction of endothelial cells is thought to be the initial step in AS [46]. The permeability of the intima and adhesion of leukocytes increase when vascular endothelial cells (VECs) are damaged, which accelerates the development of AS and stimulates thrombus formation [47].

The ferroptotic cascade in VECs is activated by diverse molecular triggers with distinct mechanisms. Bai et al. found that oxidized low-density lipoprotein (ox-LDL) causes ferroptosis in mouse aortic endothelial cells, which may be associated with increased ROS production. Ferroptosis inhibition increased SLC7A11 and GPX4 levels, and reduced iron accumulation, lipid peroxidation, and adhesion molecule expression [48]. Chen et al. found that 1-palmitoyl-2-glutaroyl-sn-glycero-3-phosphocholine (PGPC), a component of oxidized phospholipids in atherosclerotic plaques, decreased GPX4 and GSH levels while increasing FABP3 expression in human umbilical vein endothelial cells (HUVECs) via the CD36 receptor [49,50]. In addition to oxidized phospholipid-mediated initiation, metabolism-driven iron release also activates ferroptosis. For example, Meng et al. demonstrated that HMOX1 upregulation promoted ferroptosis in diabetic human endothelial cells [51]. Besides HMOX1-mediated iron release, other metabolism-driven factors, such as hormonal deficiency, have also been increasingly recognized to trigger ferroptosis. Lv et al. showed that ovariectomized mice exhibited accelerated AS progression with elevated lipid peroxidation and iron accumulation. They further found that estradiol inhibited ferroptosis by preventing mitochondrial dysfunction and activating the NRF2/GPX4 pathway [52]. Zhu et al. reported that LOX-1, a markedly upregulated receptor in the vascular wall during atherogenesis, activates cGAS-STING signaling to increase NCOA4 expression, which in turn suppressed GPX4 and SLC7A11 expression, thereby driving ferroptosis in HUVECs [53]. These triggers, including oxidized lipoprotein, oxidized phospholipids, metabolic factors, and receptor regulation, represent endogenous disease mechanisms with endothelial-specific features. The molecular execution pathways have been elucidated and validated through various pharmacological studies.

The essential pathways executing ferroptosis in endothelial cells usually involve three principal axes, with notable cell type-specific modifications. The disruption of SLC7A11/GSH/GPX4 signaling, the amino acid antioxidant defense axis, lipid peroxidation and iron metabolic disorder contribute to the increased ferroptosis and then AS development. Bai et al. demonstrated that ferrostatin-1 upregulated SLC7A11 and GPX4 expression and decreased the iron content [48]. Xiang showed that N-acetylneuraminic acid (Neu5Ac) inhibited the XC-/GSH/GPX4 pathway to promote ferroptosis. They also observed that the mRNA levels of SLC7A11 and SLC3A2 increased, but their protein levels decreased. Mechanistically, Neu5AC promoted the binding of SLC3A2 to ubiquitin, which induced SLC3A2 degradation mediated by P62, resulting in the reduced clearance of peroxidized lipids in VECs [54]. In summary, the modulation of amino acid antioxidant defenses, lipid peroxidation, or iron metabolism can effectively regulate ferroptosis, thereby influencing atherosclerosis development.

Besides the core regulatory pathway, other modulators adjust execution through alternative mechanisms. Wang et al. observed that icariin alleviated ox-LDL-induced ferroptosis by increasing TRPML1 expression to promote transcription factor EB (TFEB) dephosphorylation and translocation into the nucleus, where it regulated the expression of autophagic genes, thereby decreasing ferroptosis through enhanced mitophagy to promote the clearance of damaged mitochondria and thus reduce the mitochondrial production of free radicals, rather than through direct SLC7A11/GSH/GPX4 modulation [55]. Gao et al. found that sestrin 1 (SESN1) overexpression inhibited ferroptosis by activating p21 [56]. These interventions demonstrate that ferroptosis modulation extends beyond the three principal axes to encompass autophagic and metabolic regulatory nodes.

#### 3.1.3. Vascular Smooth Muscle Cells

Vascular smooth muscle cell (VSMC) death in advanced atherosclerotic lesions precipitates profound vascular inflammation, coupled with the depletion of collagen and extracellular matrix components, ultimately resulting in the rupture of atherosclerotic plaques [57]. The osteogenic transdifferentiation and mineralization of VSMCs play an important role in AS, which results in vascular calcification and stiffening [57,58].

The amino acid antioxidant defense axis plays an important role in VSMC ferroptosis. Chen et al. demonstrated that Yes-associated protein 1 (YAP1) stimulated glutaminase 1 (GLS1) expression to promote glutamate (Glu) production for glutathione (GSH) synthesis and to increase GPX4 activity, eventually inhibiting VSMC ferroptosis [59]. Zhang et al. showed that echinatin activated Nrf2 to increase the expression of the catalytic subunit of glutamate cysteine ligase (GCLC) and modulatory subunit of glutamate cysteine ligase (GCLM) in VSMCs to promote GSH synthesis and finally inhibit VSMC ferroptosis, relieving AS [60].

**Figure 3:**
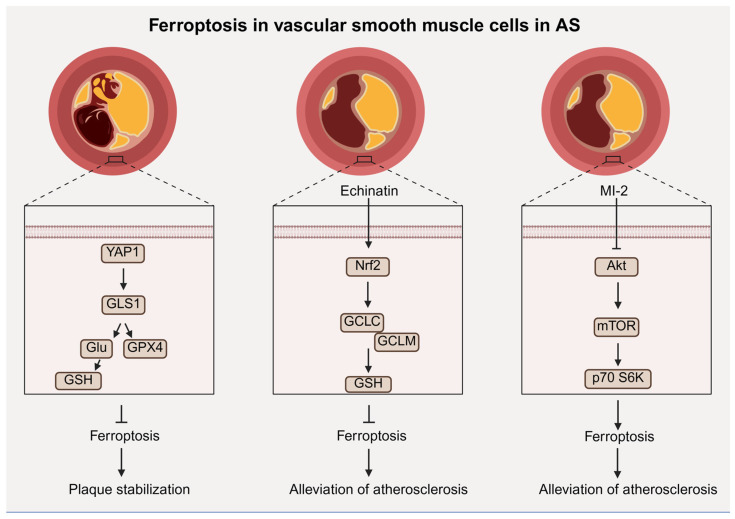
The mechanisms of ferroptosis in vascular smooth muscle cells (VSMCs) during atherosclerosis (AS) development. YAP1 increases GLS1 level to promote Glu generation for the further production of GSH and stimulation of GPX4 activity, eventually inhibiting ferroptosis and then stabilizing the atherosclerotic plaques. Echinatin stimulates Nrf2 to upregulate GCLC and GCLM levels in VSMCs to increase GSH production, eventually inhibiting VSMC ferroptosis and relieving AS development. MI-2 inhibits the Akt/mTOR/p70 S6K pathway to activate the autography-induced ferroptosis of VSMCs, inhibiting the early development of AS. In the figure, straight arrows (→) represent activation or positive regulation, whereas T-shaped arrows (⊣) represent inhibition or negative regulation. Abbreviation: YAP1, Yes-associated protein 1; GLS1, Glutaminase 1; Glu, Glutamate; GSH, Glutathione; GPX4, Glutathione Peroxidase 4; Nrf2, Nuclear factor erythroid 2-related factor 2; GCLC, catalytic subunit of glutamate cysteine ligase; GCLM, modulatory subunit of glutamate cysteine ligase; MI-2, (compound name, no expansion); mTOR, mechanistic target of rapamycin; p70 S6K, p70 S6 kinase. Created in BioRender. Yiyang, C. (2026) https://BioRender.com/iew5bao.

Other regulatory pathways also contribute to VSMC ferroptosis. You et al. demonstrated that ferrostatin-1 (Fer-1) can inhibit VSMC ferroptosis by activating the NRF2/FSP1 pathway instead of the p53/SCL7A11/GPX4 axis [61]. Moreover, Yan et al. found that MI-2 can attenuate AS by inducing VSMC ferroptosis [62]. Mechanistically, MI-2 inhibited the Akt/mTOR/p70 S6K pathway to activate the autography-dependent ferroptosis of VSMCs, decreasing contractility and inhibiting the formation of new intima and early development of atherosclerosis [62]. Ferroptosis of VSMCs plays a dual role in AS development, providing a novel approach for finding therapeutic strategies for AS (Figure 3).

#### 3.1.4. Conclusions and Perspectives

Ferroptosis operates as a cell type-specific driver of atherosclerotic progression across VECs, VSMCs, and macrophages, with similar principal axes governing its execution in each cellular context. The core regulatory axes of ferroptosis are subjected to multi-axis coordination, with such integrated regulation yielding diverse inflammatory consequences, depending on the cell type and disease stage. While endothelial ferroptosis initiates barrier dysfunction and leukocyte adhesion, and smooth muscle cell death contributes to plaque destabilization through matrix degradation, macrophage ferroptosis represents the immunological linchpin that accelerates disease evolution. These innate immune cells function as both sensors and amplifiers of the ferroptotic microenvironment, translating lipid peroxidation and iron dysregulation into inflammatory signals that promote necrotic core formation and plaque vulnerability. The central role of macrophage ferroptosis is underscored by consistent evidence that the pharmacological inhibition of macrophage ferroptosis effectively attenuates atherosclerotic lesion development and stabilizes plaque architecture. This important position of macrophages suggests that macrophage-directed ferroptosis suppression represents the most promising therapeutic approach, as targeting these cells can simultaneously interrupt lipid-driven inflammation, preserve efferocytosis efficiency, and prevent the formation of unstable necrotic cores.

### 3.2. The Role of Ferroptosis in Pulmonary Hypertension

Pulmonary hypertension (PH) is marked by a normal elevation of the pulmonary artery pressure [63]. It is not rare; in fact, it affects approximately 1% of the global population. Approximately 10% of people over 65 years of age are affected by PH [64]. The updated diagnostic hemodynamic criteria are an average pulmonary artery pressure > 20 mm Hg and pulmonary vascular resistance > 2.0 Wood units [65]. The main symptom of pulmonary hypertension is progressive exertional dyspnea, which is frequently associated with fatigue and exhaustion [66]. Despite the many therapeutic options that have been explored over the past two decades, treatment methods are still largely palliative [66]. Further investigation of the key factors regulating PH is needed to develop novel therapies for PH. Recently, many researchers found that ferroptosis plays a role in PH.

#### 3.2.1. Pulmonary Artery Endothelial Cells

Pulmonary artery endothelial cell (PAEC) injury is one of the initial events in PH progression, and PH patients have injured and/or dysfunctional PAECs [67,68].

The essential mechanism of ferroptosis in PAECs is based on three principal axes with extensive cross-regulation. Xie et al. observed increased NOX4 expression and decreased GPX4 and FTH1 levels in PAECs from the MCT-induced PH group, suggesting that ferroptosis was driven by the disruption of both the amino acid antioxidant defense axis and iron metabolism axis. Moreover, PAEC ferroptosis activated the HMGB1/TLR4 NLRP3 signaling pathway to trigger inflammatory responses and promote PH development [69]. An et al. found that pulmonary microvascular endothelial cells (PMVECs) engulfed erythrocytes under hypoxic conditions, causing increased ROS production and lipid peroxidation with decreased GPX4 and SLC7A11 levels, suggesting erythrophagocytosis coordinately disrupts the amino acid antioxidant defense axis and lipid peroxidation axis to promote PMVEC ferroptosis. The expression of TMEM16F, which was associated with various forms of cell death, including ferroptosis, was increased in the lungs of mice with hypoxia-induced PAH. Future experiments designed to study the detailed mechanism are required [70].

Other regulatory pathways also contribute to ferroptosis. For example, Liao et al. showed that PRDX6, a peroxiredoxin (PRDX) family member, was downregulated in the MCT-induced PH group. PRDX6 overexpression inhibited PAEC ferroptosis by inhibiting the HMGB1/TLR4/NLRP3 inflammasome signaling pathway, improving PH [71].

#### 3.2.2. Macrophages

The injured ECs in individuals with PH can influence macrophages through factors such as high mobility group box 1 (HMGB1) [68,69].

Liao et al. found that PRDX6 was downregulated in individuals with PH, and the expression levels of HMGB1, TLR4 and NLRP3 inflammasome markers were lower in rats from the MCT+LV-PRDX6 group than in those from the MCT+LV-Ctr group. These results implied that PRDX6 overexpression attenuated HMGB1 release from PAECs, subsequently suppressing TLR4/NLRP3 inflammasome activation and inflammatory cytokine secretion in macrophages. PRDX6 overexpression inhibits ferroptosis in vivo by inhibiting the HMGB1/TLR4/NLRP3 inflammasome pathway to attenuate PH development [71].

#### 3.2.3. Pulmonary Artery Smooth Muscle Cells

**Figure 4:**
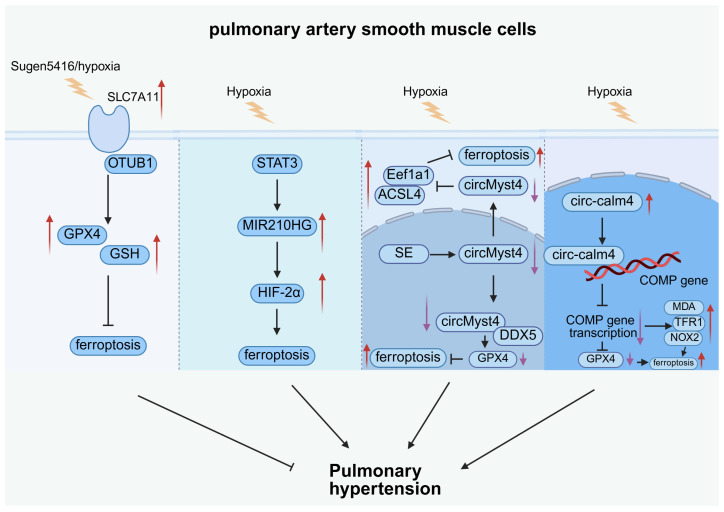
The roles and mechanisms of ferroptosis of pulmonary artery smooth muscle cells in pulmonary hypertension (PH). Sugen5416/hypoxia increases the expression of SLC7a11, which binds to OTUB1 to increase the levels of GPX4 and GSH to decrease ferroptosis, preventing PAH. STAT3 increases the level of MIR210HG in PASMCs in individuals with hypoxic pulmonary hypertension to suppress the degradation of HIF-2α, leading to the activation of ferroptosis, which is induced by autography, and then the development of PH. On the one hand, the downregulated SE-driven circMyst4 in individuals with PH under hypoxic conditions decreases its interaction with DDX5 to reduce the GPX4 level in the nucleus, increasing ferroptosis and promoting PH development. On the other hand, decreased circMyst4 expression promotes the interaction between Eef1a1 and ACSL4 in the cytoplasm of PASMCs to increase ferroptosis. Hypoxia increases the circ-calm4 level to form circR-loops with the COMP gene promoter region in the nucleus to suppress COMP gene transcription; increase the levels of TFR1, NOX2, MDA; inhibit GPX4 expression; and finally activate PASMC ferroptosis and promote PH development. In the figure, red upward arrows (↑) indicate upregulation (increase), and purple downward arrows (↓) indicate downregulation (decrease) of the indicated molecules or signaling events. Abbreviation: OTUB1, ubiquitin aldehyde binding 1; GPX4, Glutathione Peroxidase 4; GSH, Glutathione; circ-calm4, circular calmodulin 4; COMP, Cartilage oligomeric matrix protein; TFR1, transferrin receptor 1. Created in BioRender. Yiyang, C. (2026) https://BioRender.com/s9qhjp0.

In pulmonary hypertension, the suppression of VSMC apoptosis coupled with an increased proliferative capacity induces arterial wall stiffening, thereby increasing vascular resistance and exacerbating PH progression [68]. In recent research, the ferroptosis of pulmonary artery smooth muscle cells (PASMCs) was found to play an important role in PH (Figure 4), which provided potential opportunities to search for new therapies.

The ferroptotic cascade in PASMCs is initiated by diverse molecular triggers. In PH, hypoxic conditions and Sugen5416 exposure represent primary exogenous triggers that regulate PASMC ferroptosis [72,73,74,75].

The principal axes of ferroptosis in PASMCs represent the main execution mechanism. First, the modulation of the amino acid antioxidant defense axis (SLC7A11/GSH/GPX4) determines ferroptosis sensitivity. Hu et al. demonstrated that SLC7A11, which is upregulated in PAH patients and experimental models, promoted GPX4 and GSH synthesis by binding to ubiquitin aldehyde binding 1 (OTUB1) to stabilize SLC7A11 itself, thereby decreasing ferroptosis and preventing PAH [72,73,74]. Second, the activation of the lipid peroxidation axis also contributes to ferroptosis execution. He et al. found that a new circRNA, mmu_circ_0000505 (circMyst4), was downregulated in individuals with PH under hypoxic conditions and had inhibitory effects on hypoxia-induced ferroptosis. Mechanistically, in the nucleus, circMyst4 combined with DEAD box helicase 5 (DDX5) to promote GPX4 mRNA processing mediated by DDX5 to increase the GPX4 level and then inhibit ferroptosis, attenuating hypoxia-induced PH. In addition, circMyst4 suppressed the interaction of the eukaryotic translation elongation factor 1 alpha 1 (Eef1a1) and ACSL4 to decrease ferroptosis in the cytoplasm of PASMCs, suggesting that circMyst4 exerted dual anti-ferroptotic effects by coordinately regulating both the amino acid antioxidant defense axis and the lipid peroxidation axis through ACSL4 suppression. Moreover, a superenhancer (SE) was demonstrated to drive the generation of circMyst4. Their findings may show that SE-driven circMyst4 suppressed ferroptosis in PASMCs to improve hypoxia-induced PH [76].

Besides core regulatory pathways, other modulators also regulate ferroptosis via secondary mechanisms. Wang et al. found that the expression of the lncRNA MIR210HG was elevated in a hypoxic pulmonary hypertension (HPH) patient. They showed that STAT3 bound to the promoter region of MIR210HG to promote the transcription of MIR210HG in hypoxic PASMCs to inhibit the degradation of HIF-2α, a key signal that activates ferroptosis, causing the activation of autography-induced ferroptosis and a change in PASMC phenotypes in the early stage of HPH to promote HPH development [75]. Liu et al. observed that the expression of circ-calmodulin 4 (circ-calm4) was increased in the nuclei of hypoxic PASMCs to stimulate ferroptosis. Mechanistically, circ-calm4 formed circR-loops with the promoter region of the COMP gene, a downstream effector of circ-calm4, in the nucleus to inhibit COMP gene transcription and increase levels of TFR1, NOX2, MDA, and ferrous iron and inhibit GPX4 expression, eventually stimulating PASMC ferroptosis. These results provided a new therapeutic target for PH [77].

#### 3.2.4. Conclusions and Perspectives

Ferroptosis drives pulmonary hypertension progression through distinct cell type-specific mechanisms, with pulmonary artery endothelial cells and smooth muscle cells serving as primary pathological substrates. However, macrophages function as the critical immunological intermediary that amplifies and sustains the ferroptotic cascade. Positioned at the nexus of endothelial injury and vascular inflammation, macrophages translate ferroptotic signals into systemic inflammatory responses, with their activation state directly governing disease progression. This central immunomodulatory role establishes macrophage-directed interventions as a strategic therapeutic strategy capable of disrupting the pathological dialog between ferroptosis and vascular remodeling in pulmonary hypertension.

### 3.3. The Role of Ferroptosis in Aneurysm

Aortic aneurysm (AA) is a silent and progressive degenerative disease characterized by the localized dilation of the aorta, often remaining asymptomatic until a catastrophic and highly fatal rupture. To date, no pharmacological therapy has been proven to halt aneurysm growth, a gap that stems from an incomplete understanding of the cellular mechanisms driving the relentless weakening of the aortic wall. The progression of AA is underpinned by a triad of pathologies: chronic inflammation, degradation of the extracellular matrix, and a profound loss oVSMCs [78]. Emerging evidence positions ferroptosis as an important executioner in this process, driving a “ferroptosis–inflammation circuit” that bridges the gap between innate immune activation and structural collapse. Notably, studies have shown that apoptosis and necrosis inhibitors are unable to prevent the death of vascular smooth muscle cells caused by smoking, while ferroptosis inhibitors can completely rescue the cells. This indicates that they play a dominant role in response to specific triggers [79]. Consistently, Hu et al. [80] reported that the dihydrolipoamide dehydrogenase (DLD) gene coordinates the interactions between necrosis, apoptosis, and mitophagy, supporting the multi-factor cell death network in AA, where ferroptosis is the main pathogenic executor.

The development of an aneurysm involves a complex interplay between resident aortic cells and infiltrating immune cells. This creates a pathological cascade where each cell type presents a unique opportunity for targeted therapeutic intervention.

#### 3.3.1. Macrophages and Neutrophils

These cells establish a state of severe and persistent oxidative stress. Work by researchers like Zheng et al. and Packer has shown that such conditions promote the iron accumulation necessary for ferroptosis [81,82]. Single-cell RNA sequencing analysis has revealed a significant upregulation of ferroptosis-related genes in infiltrating macrophages from human abdominal aortic aneurysm (AAA) tissue, positioning these cells as key drivers of the ferroptotic microenvironment [83]. The primary biochemical execution within this niche involves the catastrophic collapse of the SLC7A11/GSH/GPX4 antioxidant axis, where the accumulation of free iron catalyzes the non-enzymatic Fenton reaction to generate lipid peroxides. This initiation is integrated within the mitochondria, where excessive mitochondrial ROS serves as the early oxidative pressure that sensitizes resident cells to iron-dependent death. Notably, a disease-specific trigger in AA is the release of neutrophil extracellular traps (NETs), which act as a potent immunological priming signal by depleting mitochondrial GSH in VSMCs via SLC25A11 inhibition, thereby lowering the threshold for lipid peroxidation in the aortic wall [84,85].

In contrast to this core execution machinery, several secondary regulatory nodes serve as “volume knobs” to modulate the ferroptotic sensitivity of the microenvironment. The specialized lipid mediator Resolvin D1 (RvD1) functions as a critical secondary regulator by mitigating macrophage ferroptosis through FPR2 signaling and Nox2 inhibition, thereby providing a signal to resolve aortic inflammation [86]. Similarly, exogenous interventions such as tea polyphenol-derived carbon dots and ROS-responsive nanoparticles loaded with selenomethionine provide auxiliary antioxidant support to neutralize the hostile microenvironment [87,88]. Furthermore, mesenchymal stem cell-derived extracellular vesicles (MSC-EVs) offer a context-specific modulatory mechanism to specifically inhibit NET-induced death [84].

Consequentially, the execution of ferroptosis in this niche completes the feedback loop by releasing DAMPs that activate the NLRP3 inflammasome, triggering a self-amplifying cycle of immune cell recruitment and structural damage.

#### 3.3.2. Vascular Smooth Muscle Cells

VSMC is the central target of ferroptosis, and its progressive loss is the direct step that causes the aortic wall failure defining an aneurysm. This vulnerability is heightened by several mechanisms. The initiation of VSMC ferroptosis is primarily driven by exogenous risk factors, including cigarette smoke extract (CSE), oxidized low-density lipoprotein (oxLDL), and environmental pollutants like F-53B [79,89,90]. The primary execution of VSMC death is governed by the collapse of the canonical GPX4 axis and mitochondrial bioenergetic failure, which is characterized by mitochondrial fragmentation and an increased membrane density [79]. Importantly, the peroxidation of mitochondrial cardiolipin serves as an essential terminal step that connects mitochondrial injury to the loss of the VSMC contractile phenotype [18]. This entire process centers on mitochondrial dysfunction and bioenergetic failure. In the early phase of ferroptotic pathway activation, impaired oxidative phosphorylation and mitochondrial lipid peroxidation drive the pathological phenotypic switching of VSMCs from a contractile to a synthetic, pro-inflammatory state, compromising the structural integrity of the aortic media. As oxidative stress intensifies and antioxidant defenses collapse, cells progress to ferroptotic death [91]. This phenotypic transformation significantly weakens the tensile strength of the aortic media, predisposing it to dilation. The direct inhibition of ferroptosis with compounds like ferrostatin-1, a well-documented inhibitor that has been reviewed by Scarpellini et al., has been shown to alleviate AAA formation by activating the SLC7A11/GPX4 axis in various studies [92].

Beyond this core machinery, several secondary modulatory factors fine-tune the VSMC ferroptotic threshold. The protective protein Heat Shock Protein Family B (Small) Member 1 (HSPB1) acts as a secondary regulator by inhibiting Dipeptidyl Peptidase 4 (DPP4) activity to suppress oxLDL-induced death, while the glycosphingolipid ganglioside GM3 acts as an auxiliary node to restrict aberrant cellular iron uptake [89,93]. Additionally, specific signaling molecules like miR-361-5p adjust the expression of iron-handling proteins to adapt to chronic injury [94]. These secondary factors represent targetable nodes that can amplify or dampen the primary ferroptotic signals. Notably, the dual roles of ferroptosis in VSMCs highlight that, while its canonical execution leads to medial decay, its fine-tuning by these modulators dictates the rate of aneurysm progression and the eventual risk of rupture.

#### 3.3.3. Conclusions and Perspectives

Ferroptosis mediates the pathological weakening of the aortic wall through a destructive dialog between infiltrating innate immune cells and structural vascular smooth muscle cells. While the progressive loss of VSMCs is the ultimate driver of aortic dilation and rupture, neutrophils and macrophages function as the primary pro-ferroptotic orchestrators. The identification of NET-induced VSMC ferroptosis represents a critical immunological trigger that links acute immune activation to structural failure. This “immune-to-structural” pathological cascade suggests that the most innovative therapeutic frontier for aneurysms lies in immunometabolic resolution using specialized mediators like Resolvin D1 or ROS-responsive delivery systems to quench the hostile microenvironment and preserve the VSMC contractile phenotype.

### 3.4. The Role of Ferroptosis in Aortic Dissection

Aortic dissection (AD) is a hyperacute and life-threatening cardiovascular catastrophe, representing an acute form of aortic wall failure. This contrasts with the slow, degenerative process of an aortic aneurysm (AA), yet both diseases share a common core pathology. The progressive loss ofVSMCs is a hallmark of the medial degeneration that predisposes the aorta to failure, and ferroptosis has emerged as a crucial mechanism responsible for this critical cell loss. In AD, this process involves a destructive interplay between different cell types, each contributing to the catastrophic failure of the aortic wall.

#### 3.4.1. CD4^+^ T Cells

The degenerative process of the aortic wall is underpinned by chronic inflammation, which involves the infiltration of immune cells that orchestrate a pro-ferroptotic environment. While multiple immune cells are involved, Li et al. have recently highlighted a key role for CD4^+^ T cells. The initiation of ferroptosis in CD4^+^ T cells in the context of acute Stanford type-A AD (ATAAD) is primarily driven by the metabolic reprogramming of the systemic inflammatory microenvironment. The sequence of events initiates with the sudden rupture of the aortic wall, which triggers an intense sterile inflammatory response and shifts the lipid profile of the circulatory environment. This pathological shift induces the acute upregulation of the lipid scavenger receptor CD36 on CD4^+^ T cells, which facilitates the excessive uptake of fatty acids (FAs), particularly palmitic acid. The primary biochemical execution of T cell ferroptosis is then driven by a synergistic failure—the massive influx of lipids leads to the hyperactivation of ACSL1 and the subsequent accumulation of intracellular iron and MDA, which overwhelms the cell’s primary antioxidant defenses. Crucially, this execution is centered on mitochondrial damage. Transmission electron microscopy of samples from ATAAD patients reveals ferroptosis-specific morphological traits, including the development of mitochondrial vacuoles, an increased membrane density, and the loss of mitochondrial cristae. These structural defects are accompanied by a compromised mitochondrial transmembrane potential (ΔΨm) and reduced mitochondrial mass, effectively establishing a state of bioenergetic failure within the adaptive immune compartment. Notably, whole transcriptome profiling suggests unique crosstalk within these cells, where ferroptosis pathways are more significantly enriched than apoptosis or necrosis, positioning it as the dominant driver of T cell depletion in acute aortic injury [95].

#### 3.4.2. Vascular Smooth Muscle Cells

VSMCs are key target cells in the abnormal aortic environment, and their death, which also involves ferroptosis, directly leads to aortic wall rupture. The initiation of this process is triggered by intense oxidative stress and clinical risk factors such as cigarette smoke [79]. The primary executioner of ferroptosis in AD is the unique epitranscriptomic suppression of the core machinery; specifically, the upregulation of methyltransferase-like 3 (METTL3) promotes the m6A modification and subsequent silencing of SLC7A11 and FSP1 [96]. This collapse of the primary antioxidant shield, coupled with the Hypoxia-Inducible Factor 1-Alpha (HIF-1α)/Heme Oxygenase 1 (HMOX1)-mediated release of labile iron, induces the Fenton reaction and drives rampant lipid peroxidation. This execution is centered on mitochondrial dysfunction. In the early phase of ferroptotic pathway activation, ROS-induced damage facilitates the rapid pathological phenotypic switching of VSMCs from a contractile to a synthetic state, compromising the structural integrity of the aortic media. As the pathway progresses and antioxidant defenses collapse, cells undergo ferroptotic death, and the combined effects of phenotypic transformation and cell death drastically reduce the tensile strength of the aorta [97,98,99].

Beyond these primary execution axes, several secondary regulatory factors fine-tune the VSMC ferroptotic threshold in AD. The histone acetyl transferase P300 acts as a modulatory node. Its deficiency, which is often induced by ferroptotic stress, promotes the binding of HIF-1α to P53, which further triggers HMOX1 overexpression and amplifies the iron-dependent death signal [100].

#### 3.4.3. Conclusions and Perspectives

**Figure 5:**
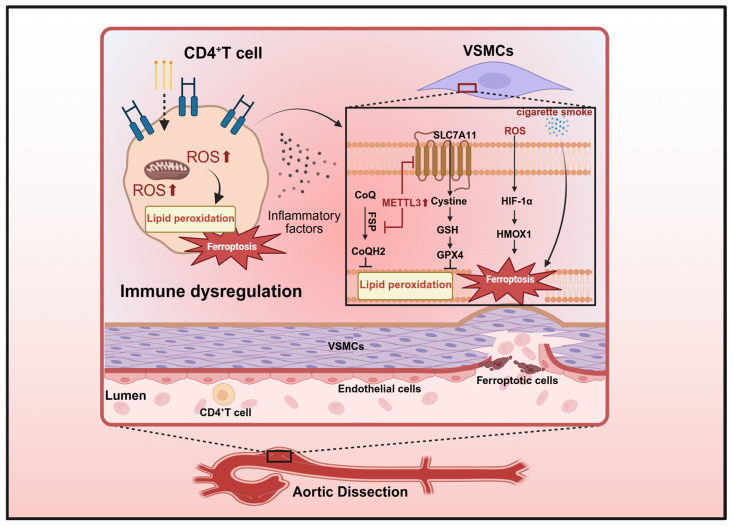
The roles and mechanisms of ferroptosis of CD4^+^ T cells and VSMCs in aortic dissection (AD)**.** CD4^+^ T cell and VSMC ferroptosis synergistically promotes the development of AD. On the left, ferroptosis pathways mediated by the lipid scavenger receptor CD36 are enriched in CD4^+^ T cells, leading to increased reactive oxygen species (ROS) production and lipid peroxidation. This causes ferroptosis and immune dysfunction in these cells, releasing inflammatory factors that further exacerbate damage to the aortic wall. On the right, VSMCs stimulated by cigarette smoke and other factors generate ROS, activating the HIF-1α/HMOX1 axis. Meanwhile, the methyltransferase METTL3 is upregulated, suppressing the expression of SLC7A11 and FSP1, resulting in downregulation of the anti-ferroptosis proteins GPX4, SLC7A11, and FSP1. This triggers lipid peroxidation and ferroptosis. Together, these processes lead to a loss of VSMCs in the aortic media and structural damage to the vascular wall, ultimately driving the occurrence of AD. Abbreviation: VSMCs, Vascular Smooth Muscle Cells; ROS, Reactive Oxygen Species; SLC7A11, Solute Carrier Family 7 Member 11; CoQ, Coenzyme Q; FSP, Ferroptosis Suppressor Protein; CoQH2, Reduced Coenzyme Q; METTL3, Methyltransferase Like 3; GSH, Glutathione; GPX4, Glutathione Peroxidase 4; HIF-1α, Hypoxia-Inducible Factor 1-alpha; HMOX1, Heme Oxygenase 1. Created in BioRender. Pengyan, Z. (2026) https://BioRender.com/hm2mtcv.

AD represents a hyperacute synergistic failure of adaptive immunity and vascular structural integrity, with ferroptosis emerging as a central molecular executioner. A paradigm-shifting insight in AD research is the role of CD36-mediated ferroptosis in destabilizing CD4^+^ T cell homeostasis, as shown in Figure 5, which cripples the vascular immune defense and exacerbates medial degeneration. This positions AD not merely as a mechanical event, but as a catastrophe of dysfunctional immune–vascular crosstalk. Future research should prioritize the epitranscriptomic regulation (such as the METTL3-mediated m6A modification) of the ferroptosis–inflammation axis, offering a novel strategy to stabilize both immune cell function and VSMC architecture in patients with this life-threatening condition.

## 4. Ferroptosis in Cerebrovascular Diseases

### 4.1. The Role of Ferroptosis in Stroke

Globally, stroke represents a major health challenge, ranking among the top contributors to both mortality and permanent disability [101]. Ischemic stroke, which accounts for over 80% of cases, occurs when a cerebral artery is occluded. Although current reperfusion strategies, namely, thrombolysis and mechanical thrombectomy, are transformative for eligible patients, their clinical impact is severely hindered by a narrow therapeutic window and limited patient accessibility. Crucially, the very act of restoring blood flow, while essential, paradoxically triggers a secondary wave of cellular damage known as ischemia–reperfusion (I/R) injury, which significantly contributes to the final infarct volume [102,103,104]. This has driven an urgent search for neuroprotective agents that can shield the brain tissue from this damage [105]. As it is morphologically and biochemically distinct from traditional programmed cell death (PCD) modalities, ferroptosis in stroke is defined by mitochondrial shrinkage and rampant lipid peroxidation [106]. Furthermore, within the landscape of diverse cell death modalities, ferroptosis, which is driven by iron-mediated lipid peroxidation, has been identified as a critical factor in stroke pathogenesis. Its pathogenic weight is particularly pronounced during the reperfusion phase, where it emerges as a predominant executioner alongside necroptosis, often surpassing early-stage apoptosis in governing the expansion of the ischemic penumbra [106,107]. The process of ferroptosis begins with the accumulation of iron and a breakdown of cellular redox protection, culminating in rampant lipid peroxidation and the loss of membrane integrity [7,23]. This process is not confined to one cell type but permeates the entire neurovascular unit, creating a complex injury landscape.

Crucially, ferroptosis acts as a sterile inflammatory catalyst, converting intracellular metabolic collapse into an immunogenic signal through the release of DAMPs. This mechanism triggers a deleterious inflammatory response that spreads across the neurovascular unit. Within this environment, the ferroptotic death of diverse cell populations, such as neurons, glia, and endothelial cells, collectively governs the advancement of cerebral damage and the shifting neuroinflammatory landscape.

#### 4.1.1. Neurons

As the fundamental functional units of the central nervous system, neurons are the primary and most critical victims of ferroptosis following a stroke. Their pronounced susceptibility is intrinsically linked to their physiological characteristics, particularly their high metabolic activity, which predisposes them to elevated levels of oxidative stress and a reliance on iron-dependent enzymatic systems. This inherent vulnerability makes them a focal target for the ferroptotic cascade when intracellular iron homeostasis is disrupted, and antioxidant defenses collapse during ischemia–reperfusion (I/R) injury. From a clinical perspective, the widespread death of neuronal populations via ferroptosis is devastating, as it forms the direct cellular basis for the profound neurological deficits, including motor, sensory, and cognitive impairments, observed post-stroke [108].

Their susceptibility is linked to high metabolic activity, oxidative stress, and a reliance on iron-dependent systems like the tau protein [109].

The sequence of events initiates during the I/R phase, where the restoration of flow triggers the TLR4/NF-κB pathway, causing an acute ROS burst [110]. The primary execution of death is then driven by a synergistic imbalance. While the hyperactivation of ACSL4 accelerates the remodeling of membrane phospholipids with oxidizable PUFAs [111], the cell’s primary defense, the SLC7A11/GSH/GPX4 axis, simultaneously collapses. Subsequently, this metabolic failure triggers the STAT3/HIF-1α/PTRF axis, which increases *PLA2G4A* expression to target mitochondrial lipids, leading to mitochondrial bioenergetic failure and terminal cell death [112]. In hemorrhagic stroke, reduced lactoferrin exacerbates this process by allowing intraneuronal iron accumulation [113]. Crosstalk with other death modes is evident; for instance, TNF-α can simultaneously trigger apoptosis and ferroptosis [106], while the necroptosis inhibitor Necrostatin-1 provides cross-protection [106].

The modulatory landscape involves secondary factors. Kaempferol and brain-penetrant selenopeptides increase the activity of the Nrf2/SLC7A11/GPX4 pathway to mitigate peroxidation [114,115]. Furthermore, advanced drug delivery systems, such as engineered anti-CHAC1 exosomes designed for nose-to-brain administration [116], are being developed to specifically inhibit neuronal ferroptosis.

#### 4.1.2. Glial Cells

The impact of ferroptosis extends to the brain’s supportive glial network, where it contributes significantly to post-stroke pathology.

Mature oligodendrocytes and oligodendrocyte progenitor cells (OPCs) exhibit extreme vulnerability to ferroptotic execution, representing a primary mechanism of white matter injury (WMI) after both ischemic and hemorrhagic strokes [117]. The sequence of events initiates hyperacutely; for instance, Gu et al. utilized single-cell and spatial transcriptomics to reveal that ferroptosis is the most enriched pPCD process in individuals with hemorrhagic stroke, affecting mature oligodendrocytes as early as one-hour after injury [118]. The primary execution is governed by the PLIN2-mediated lipid remodeling axis, where the lipid storage protein Perilipin-2 (PLIN2) facilitates the catastrophic peroxidation of PUFA-PLs and subsequent myelin damage [119]. Crucially, this execution involves mitochondrial shrinkage and an increased membrane density [106]. Under these conditions, the catastrophic loss of the SLC7A11/GSH/GPX4 shield leads to irreversible structural failure of the white matter.

The modulatory landscape and feedback loop in oligodendrocytes are characterized by complex crosstalk with autophagy and inflammatory signaling. While the cytokine IL-10 serves as an endogenous secondary modulator that protects OPCs by reducing lipid ROS levels [117], the process is often exacerbated by autophagy-dependent ferroptosis (ferritinophagy), which increases the labile iron pool. Crosstalk with apoptosis is also suggested in the context of noise-induced and ischemic injury, where shared oxidative triggers activate multiple RCD pathways simultaneously [106]. Consequentially, the ferroptotic failure of oligodendrocytes releases LCN2-positive signals, which is hypothesized to establish a putative feedback loop by recruiting neurotoxic microglia [118]. This self-amplifying circuit provides a mechanistic explanation for the failure of myelin regeneration observed in post-stroke recovery.

This wave of ferroptotic cell death is not limited to oligodendrocytes, extending to other vital glial populations as well.

Astrocytes serve as critical supporters of the blood–brain barrier (BBB) and regulators of the neuronal microenvironment, but are also highly susceptible to ferroptosis [120], and their death and dysfunction significantly contribute to post-stroke pathology. Astrocytes are the only nerve cell type in the brain that stores glycogen, making them crucial for regulating glucose metabolism and supplying energy to neurons [121]. Consequently, astrocyte ferroptosis not only compromises the structural support of the neurovascular unit but also leads to an energy crisis in surrounding neurons.

The initiation phase is triggered by an ischemic insult, which upregulates N-myc downstream-regulated gene 2 (NDRG2) and subsequently activates pro-inflammatory signaling via the inhibition of the Wnt/β-catenin pathway [121]. The primary executioner in astrocytes is the loss of metabolic homeostasis. In in vitro oxygen-glucose deprivation/reoxygenation (OGD/R) models, NDRG2 overexpression has been shown to increase the labile iron and ROS levels while depleting the SLC7A11/GSH/GPX4 antioxidant shield. Crucially, this is centered on mitochondrial bioenergetic failure. As astrocytes are the main providers of glycogen and glucose-derived energy for neurons [121], their ferroptosis not only results in mitochondrial membrane rupture but also deprives surrounding neurons of metabolic support, leading to a broader energy crisis in the neurovascular unit [106].

Secondary regulatory nodes act as modulatory factors to counteract this bioenergetic collapse. Studies have shown that activating the Nrf2 pathway can elicit robust neuroprotection by modulating the OXPHOS/NF-κB/ferroptosis axis [122]. In preclinical models, natural compounds such as 11-keto-β-boswellic acid (KBA) and Z-guggulsterone (Z-GS) synergistically restore the level of the iron storage protein Fth1 to suppress ferroptosis [123]. Consequentially, the terminal stage of astrocyte ferroptosis completes the feedback loop via the release of large amounts of HMGB1. This putative circuit is proposed to activate endothelial cells and increase BBB permeability, potentially allowing the infiltration of peripheral immune cells, which further exacerbates neuronal loss.

The brain’s innate immune cell population, microglia, exhibits nuanced and diverse functions in post-stroke pathology. They not only exacerbate cerebral injury but also represent a promising avenue for clinical treatment. As the primary responders to brain injury, microglia are rapidly activated and are central to the neuroinflammatory cascade. Their response is often dual-natured: they can adopt a neuroprotective M2 phenotype involved in debris clearance and repair, or a neurotoxic M1 phenotype that releases pro-inflammatory cytokines like TNF-α. Recent evidence suggests that ferroptosis is also involved in its pathological process.

Microglia contribute to damage by inducing ferroptosis in other neural cells. Activated microglia release ROS and other toxic substances that create a hostile, pro-ferroptotic environment. For instance, specific interactions between LCN2-positive microglia and oligodendrocytes have been shown to induce oligodendrocyte ferroptosis, contributing to neurological deficits after hemorrhagic stroke [118]. Furthermore, microglia can kill stressed-but-viable neurons through a process called phagoptosis, where microglial-derived oxidants induce reversible stress signaling in neurons, marking them for engulfment and destruction [124].

More recent studies have revealed that microglia not only cause ferroptosis but also undergo this process themselves, which often occurs in conjunction with another inflammatory cell death pathway, pyroptosis [125]. The sequence of events initiates with the activation of the HMGB1/ALPK1 axis, where the novel pattern recognition receptor alpha-kinase 1 (ALPK1) is most highly upregulated in microglia. The primary execution machinery involves a groundbreaking mechanistic crosstalk between ferroptosis and pyroptosis. ALPK1 drives microglial pyroptosis via the NF-κB/NLRP3 pathway and simultaneously promotes ferroptosis via the JAK2/STAT3 axis [125]. This represents a putative crosstalk mechanism where the activation of the pyroptotic inflammasome facilitates ferroptotic execution, creating a hybrid form of inflammatory death [106,125]. Furthermore, exosomal miRNA (Novel-3) from macrophage-derived foam cells has been identified as a specific trigger of this circuit in atherosclerosis-associated stroke [126].

The modulatory landscape and feedback loop in microglia involve both neurotoxic recruitment and the elimination of stressed cells. Secondary factors, such as glycyrrhizic acid, act as inhibitors to downregulate ALPK1 and suppress this hybrid death program [125]. Similarly, engineered exosomes targeting M2 microglia provide a precise therapeutic strategy to dampen the sterile neuroinflammatory storm [127]. Ultimately, this completes the “ferroptosis–inflammation circuit,” where ferroptotic microglia release oxidants that drive phagoptosis—the killing of stressed-but-viable neurons [124]. This feedback loop is proposed to be sustained through the release of microglial-derived LCN2 and DAMPs, which are hypothesized to further sensitize the neurovascular unit to secondary waves of injury [118].

#### 4.1.3. Brain Microvascular Endothelial Cells

Brain microvascular endothelial cells, which form the blood–brain barrier (BBB), are key gatekeepers of ferroptosis. Their death is a particularly pernicious pathological event because it dismantles this protective barrier, igniting a “vicious cycle of secondary injury” [128].

The sequence of events initiates with metabolic and inflammatory stressors. For instance, studies using ApoE^−/−^ mouse models and HUVECs have shown that the metabolite Neu5Ac acts as an initial trigger, while in ischemic contexts, inflammatory ligands activate pro-oxidant signaling [54,129]. The primary execution phase is governed by the catastrophic collapse of the cell’s antioxidant machinery. Specifically, the ubiquitin-mediated degradation of the SLC3A2/SLC7A11 system serves as the primary driver, resulting in rampant lipid peroxidation and mitochondrial membrane rupture [54]. In diabetic human endothelial cell models, HMOX1 upregulation paradoxically releases excess free iron, which provides the Fenton catalyst needed to overwhelm the cell’s energy-dependent redox shield [51]. The terminal result of this execution is the loss of VEC integrity, leading to vasogenic edema and the uncontrolled infiltration of circulating iron into the brain parenchyma.

The modulatory landscape and crosstalk with other death modes further dictate the severity of BBB breakdown. In contrast to the core execution machinery, several secondary modulatory nodes fine-tune the endothelial threshold. Edaravone dexborneol, for example, acts as a protective factor in CIRI mouse models by restoring the GPX4 shield via the Nrf2/HO-1 pathway [130]. Complementing these findings, clinical and experimental evidence identifies Lipocalin-2 (LCN2) as a detrimental secondary regulator of BBB integrity; specifically, baseline serum LCN2 levels in acute ischemic stroke (AIS) patients correlate with hemorrhagic transformation, while in rats with thromboembolic stroke, LCN2 facilitates endothelial ferroptosis by triggering the HMGB1-mediated inhibition of the Nrf2/HO-1 nuclear translocation [129]. Notably, this modulatory landscape exhibits complex crosstalk with apoptosis and necroptosis, where the loss of VEC integrity facilitates the entry of TNF-α, which acts as a shared trigger of multiple death pathways [106]. Consequently, the failure of the endothelium completes the neurovascular feedback loop. This putative circuit establishes BBB leakage that allows for a massive influx of iron and peripheral cytokines, which is hypothesized to further sensitize neurons and glia to ferroptosis, thereby driving post-stroke neurodegeneration.

#### 4.1.4. Conclusions and Perspectives

In stroke pathology, ferroptosis functions as a sterile inflammatory catalyst that permeates the entire neurovascular unit. The release of immunogenic DAMPs, particularly HMGB1, converts intracellular metabolic collapse into a secondary neuroinflammatory storm that dictates the final infarct volume. The discovery of the HMGB1-ALPK1 axis provides a groundbreaking mechanistic bridge between microglial ferroptosis and pyroptosis, revealing unique crosstalk between inflammatory cell death pathways. The innovative frontier for stroke therapy lies in neurovascular unit-targeted neuroprotection utilizing engineered exosomes or brain-penetrant peptides to specifically inhibit microglial neurotoxicity while fostering an anti-ferroptotic environment for neuronal survival and white matter repair.

### 4.2. The Role of Ferroptosis in Cerebral Small Vessel Disease

Cerebral small vessel disease (cSVD) represents a slow-evolving disorder where chronic hypoperfusion leads to the development of white matter lesions and a gradual loss of cognitive ability. Recent evidence suggests that ferroptosis in brain vessel endothelial cells (BVECs) is an emerging contributing factor to cSVD progression, where the death of these cells initiates a cascade of nutrient deprivation and neuroinflammation.

#### 4.2.1. Brain Microvascular Endothelial Cells

The initiation of endothelial ferroptosis in cSVD is driven by both hemodynamic and genetic stressors. The sequence of events begins with chronic hypoperfusion inducing the endocytosis of myelin debris by BVECs or, in hereditary contexts, the presence of the NOTCH3 p.C533S mutation. Subsequently, these stressors activate pro-inflammatory signaling pathways; specifically, in human hCMEC models carrying the NOTCH3 mutation, a loss of tight junctions and diminished migratory capacity are observed [131,132]. The primary execution is governed by a catastrophic collapse of the cell’s antioxidant capacity. Mechanistically, Liu et al. [132] identified that an impairment of the VEGF/VEGFR pathway contributes to reduced activities of glutathione reductase (GR) and argininosuccinate synthase 1 (ASS1), leading to the severe depletion of the GSH pool. Given that GSH is critical for mitochondrial antioxidant defense, this metabolic reprogramming likely contributes to mitochondrial dysfunction and cellular bioenergetic failure. The sequestration of iron within BVECs, as exemplified by myelin-induced iron overload, catalyzes rampant lipid peroxidation and prevents energy-dependent iron transport across the BBB [131].

The modulatory landscape and feedback loop in the endothelium are further defined by clinical predictors and precise therapeutic targets. From a clinical perspective, Sun et al. [133] identified elevated serum ferritin levels and cerebral microbleeds as independent predictors of Vascular Cognitive Impairment (VCI) in a cohort of 255 cSVD patients, reflecting the systemic ferroptotic burden. Secondary modulatory control can be achieved using edaravone dexborneol (EDB), which has been shown to partially reverse pathology in CRISPR/Cas9-engineered CADASIL models by restoring VEGF/VEGFR-regulated GSH synthesis [132].

#### 4.2.2. Neurons and Oligodendroglial Lineage Cells

In cSVD, neurons and oligodendrocytes are the terminal victims of the compromised neurovascular unit, but through distinct mechanisms. The initiation phase in these cells is unique, which is driven by the interaction with degenerated BVECs, which induces both direct pro-inflammatory signals and indirect nutrient deprivation [132]. The primary execution phase in the oligodendroglial lineage is triggered by “iron hunger” resulting from BVEC exhaustion, leading to a failure of iron-dependent myelin synthesis enzymes and mitochondrial bioenergetic failure in OPCs [131]. Simultaneously, compromised human cerebral microvascular endothelial cells (hCMECs) directly induce neuronal ferroptosis, which is characterized by accumulated lipid droplets and oxidative damage [132]. Ultimately, this completes the circuit, as the combined failure of white matter regeneration and neuronal survival is proposed to sustain the chronic neuroinflammatory environment that drives the progression of cognitive decline.

Secondary modulatory strategies aim to bypass the failed endothelial supply chain or restore cellular defenses. For instance, the intranasal administration of holo-transferrin (hTF) to UCCAO mouse models has been shown to promote myelin regeneration by delivering essential iron directly to the white matter [131]. Similarly, EDB treatment offers auxiliary protection across both hereditary and non-hereditary cSVD by mitigating the interactions between compromised endothelial cells and surrounding neurons [132].

#### 4.2.3. Conclusions and Perspectives

In summary, cSVD represents a slow-evolving manifestation of the ferroptosis–inflammation circuit, where chronic metabolic stress within the neurovascular unit precipitates a gradual decline in cognitive ability. A distinctive pathological feature of cSVD is the synergistic failure of the “endothelial-to-oligodendrocyte” iron dialog. While endothelial ferroptotic exhaustion initiates the cascade by sequestering iron and dismantling the blood–brain barrier, the resulting mitochondrial “iron hunger” in the oligodendroglial lineage effectively halts myelin regeneration. This internal supply chain failure creates a self-propagating neuroinflammatory environment that bridges vascular dysfunction with chronic neurodegeneration.

## 5. Therapeutic Applications of Ferroptosis in Vascular Diseases

Ferroptosis serves as a critical mediator of the pathogenesis of vascular diseases. These cell type-specific susceptibilities are governed by intricate molecular mechanisms, including the antioxidant defense axes, iron metabolism pathways, and lipid peroxidation cascades, which together control whether each vascular cell type undergoes ferroptosis (Table 1).

Furthermore, different vascular cell populations, including vascular endothelial cells, neutrophils and macrophages, exhibit distinct susceptibilities to ferroptosis, with their ferroptosis contributing differentially yet synergistically to vascular disease progression. The recognition of ferroptosis as a key driver of cardiovascular and cerebrovascular pathology has spurred the development of diverse pharmacological interventions targeting ferroptosis. These agents, including small-molecule ferroptosis inhibitors, iron chelators and natural phytochemicals, have unique mechanisms to suppress lipid peroxidation, restore redox homeostasis, or modulate iron metabolism, providing significant therapeutic potential for the treatment of diverse vascular diseases (Table 2).

### 5.1. Synthetic Small-Molecule Ferroptosis Inhibitors

#### 5.1.1. Ferrostatin-1

Ferrostatin-1 (Fer-1) is the first-generation ferroptosis inhibitor identified in a high-throughput screen, and it functions primarily by inhibiting lipid peroxidation [142]. In terms of cardiac and cerebral vascular pathologies, Fer-1 exerts significant protective effects on multiple disease models. Both experimental and animal studies have demonstrated that Fer-1 attenuates atherosclerotic plaque progression and macrophage-derived foam cell generation. In ox-LDL-treated macrophages, Fer-1 reduced the iron content and lipid accumulation by upregulating the ferritin heavy chain, GPX4, and scavenger receptor class B member 1 (SCARB1) through AMPK activation, highlighting the intimate connection between ferroptosis inhibition and lipid metabolism regulation in atherosclerotic plaque development [134]. Moreover, Tuo QZ et al. found that Fer-1 protected the brain from cerebral ischemic injury by reducing neuronal ferroptosis. Studies have demonstrated that Fer-1 reduces iron accumulation, prevents neuronal loss, and improves neurological outcomes after both ischemic and hemorrhagic stroke. The compound effectively crosses the blood–brain barrier and suppresses lipid peroxidation in the infract penumbra, thereby attenuating brain injury and providing a novel therapeutic approach [135]. Recent studies have revealed that Fer-1 exerted significant suppressive effects on the development and lethal outcomes of aortic aneurysm and dissection. Through the regulation of pivotal modulators, including MEF2C and KDM5A, Fer-1 maintains aortic wall structural integrity, thereby influencing immune function, redox homeostasis and SMC phenotypic stability. The inhibition of ferroptosis by Fer-1 also altered miRNA expression, particularly by upregulating miR-361-5p and downregulating miR-3151-5p, which target pathways related to inflammation and ion homeostasis [94].

#### 5.1.2. Liproxstatin-1

Liproxstatin-1 (Lipro-1), a spiroquinoxalinamine derivative, represents another potent ferroptosis inhibitor with distinct pharmacological properties from Fer-1 [15]. Fan BY et al. revealed that Lipro-1 had superior neuroprotective efficacy compared to other antioxidants. Lipro-1 attenuated mitochondrial lipid peroxidation while simultaneously reinstating GSH, GPX4, and ferroptosis suppressor protein 1 expression, conferring protection against ferroptosis in oligodendrocytes and highlighting its prospective value in mitigating CNS diseases characterized by oligodendrocyte degeneration [143]. Zhang Z found that GPX4 expression decreased during ferroptosis in individuals with intracerebral hemorrhage, suggesting that Lipro-1 may be effective in treating cerebrovascular diseases [136].

#### 5.1.3. UAMC-3203

UAMC-3203 is a second-generation ferrostatin analog developed to overcome the pharmacokinetic limitations of Fer-1, particularly its poor plasma stability and metabolic instability. As a Fer-1 analog with improved solubility and stability, UAMC-3203 exhibits improved efficacy in inhibiting ferroptosis. In spinal cord injury (SCI), primary mechanical damage immediately disrupts neurons, blood vessels, and glial cells, initiating a secondary injury cascade over subsequent hours and days characterized by increased blood–spinal cord barrier permeability, ion imbalances, lipid peroxidation, and inflammation. Kan S et al. found that UAMC-3203 activated NRF2/HO-1 signaling to reduce ROS production, inhibiting ferroptosis and inflammation [144]. This finding proved the efficacy of UAMC-3203 treatment for cerebrovascular diseases by reducing ferroptosis and neuroinflammation.

Despite their structural diversity, Fer-1, Lipro-1, and UAMC-3203 usually regulate key modulators in the lipid peroxidation axis, the iron metabolism axis, and the amino acid antioxidant defense axis, including GSH/GPX4 and ferritin heavy chain, to suppress ferroptosis. While these multi-target interventions demonstrate efficacy in preclinical models of atherosclerosis, stroke, and aortic aneurysm, no synthetic ferroptosis inhibitors have received FDA approval for cardiovascular indications. Fer-1 and Lipro-1 remain in preclinical stages due to pharmacokinetic limitations. UAMC-3203 represents pharmacological optimization but requires further validation in large animal models before its clinical translation [106,145].

### 5.2. Iron Chelation Therapy (Deferoxamine)

Deferoxamine (DFO) is an FDA-approved iron chelator that effectively inhibits ferroptosis by binding free iron and blocking Fenton reaction-induced hydroxyl radical production [15]. Hanson LR et al. found that the intranasal administration of DFO reduced the infarct volume in individuals with ischemic stroke by chelating free iron and attenuating lipid peroxidation [137]. Furthermore, a phase II clinical trial (NCT00777140) demonstrated that a dose of 40–60 mg/kg/day DFO was safe and well-tolerated in acute ischemic stroke patients without increasing thrombolytic complications. An exploratory analysis suggested a potential neurological benefit in patients with moderate-to-severe stroke, although these findings require confirmation in larger randomized controlled trials [146]. Zhao K et al. found that desferrioxamine mesylate also improved clinical outcomes, accelerated recovery and reduced the hematoma volume in patients with intracerebral hematoma, highlighting the potential application of DFO in treating cerebrovascular diseases [138].

While DFO is approved by the FDA to treat iron overload disorders, its application in cerebrovascular diseases remains investigational. The inability to target specific tissues and the risk of systemic iron deficiency require the development of targeted delivery systems or conditionally activated chelators for cardiovascular applications [106,147,148].

### 5.3. Natural Products

#### 5.3.1. Quercetin

Quercetin (QCT), a dietary flavonoid with potent antioxidant properties, exerts anti-ferroptosis effects via multiple mechanisms, including Nrf2 pathway activation and ferritinophagy inhibition. Xiong M showed that QCT interacted with the Y71 site of NCOA4 to reduce NCOA4 expression, inhibiting the ferroptosis of oligodendrocyte progenitor cells (OPCs) [149]. Moreover, QCT significantly inhibited Id2 and transferrin expression, while it increased GPX4 and PTGS2 expression, preventing ferroptosis in OPCs [150]. These findings suggested that QCT could be a viable option for cerebrovascular diseases. Lv Y et al. demonstrated that QCT activated the NRF2/GPX4 signaling pathway mediated by KEAP1 ubiquitination to suppress endothelial cell ferroptosis, leading to the attenuation of postmenopausal atherosclerosis [139].

#### 5.3.2. Salvianolic Acid A

Salvianolic acid A (SAL-A) is a water-soluble polyphenol compound extracted from *Salvia miltiorrhiza*, a traditional Chinese medicine widely used for cardiovascular and cerebrovascular diseases. Shang YF et al. found that SAL-A ameliorated ischemic stroke and was a therapeutic molecule for the treatment of ischemic stroke. Mechanistically, SAL-A inhibited ferroptosis by activating Nrf2-related pathways [140]. The therapeutic potential of SAL-A in cerebrovascular diseases was also validated by the amelioration of intracerebral hemorrhage achieved using SAL-A. Shi Y et al. found that SAL-A activated the Akt/GSK-3β/Nrf2 signaling pathway to inhibit ferroptosis, protecting against intracerebral hemorrhage [141].

Collectively, QCT and SAL-A suppress ferroptosis through the convergent activation of the Nrf2 antioxidant pathway, offering promising therapeutic potential for vascular diseases. Despite promising preclinical data, natural products face challenges in clinical translation due to poor bioavailability and a lack of standardized dosing regimens [106].

### 5.4. Therapies Targeting the Ferroptosis–Immune Interface

Emerging evidence indicates that ferroptosis interacts bidirectionally with immune-mediated inflammation in vascular diseases, offering a mechanistic basis for immunomodulatory therapeutic approaches. In the adaptive immune response, Th1 cells exacerbate ferroptosis primarily through the secretion of IFN-γ, which inhibits the expression of SLC7A11 by activating the JAK–STAT pathway [151,152]. Tregs exert anti-ferroptotic effects by stabilizing GPX4 and suppressing lipid peroxides through the direct upregulation of GPX4 through the IL-10R–STAT3 axis and inhibition of ALOX15 by suppressing pro-inflammatory signals [117,153,154]. Conversely, ferroptotic cells actively modulate immune responses via the secretion of DAMPs like HMGB1, ATP, and oxidized phospholipids, which in turn induce dendritic cell maturation, enhance macrophage phagocytosis, and facilitate CD8^+^ T cell infiltration [155,156]. This reciprocal regulation suggests that targeting the ferroptosis–immune interface may interrupt pathological feedforward loops in AS, PAH, and aneurysm progression.

Chen L et al. found that neutrophil extracellular traps (NETs) induced VSMC ferroptosis by inhibiting the PI3K/AKT pathway in AAA [85]. Chu Z et al. revealed that hypoxia-pretreated mesenchymal stem cell-derived small extracellular vesicles (Hypo-sEVs) were enriched in miR-17-5p, which decreased the NET formation by inhibiting the TLR4/ROS/MAPK pathway [157]. These findings demonstrate that targeting the NET-mediated ferroptosis–immune axis, specifically by interrupting NET-induced PI3K/AKT inhibition via reduced NET production, represents a promising therapeutic strategy to alleviate VSMC ferroptosis and attenuate AAA progression. However, future studies are required to determine whether VSMC ferroptosis in AAA creates a self-amplifying inflammatory loop.

## 6. Conclusions

In this comprehensive review, we have systematically established that ferroptosis, a form of iron-dependent regulated cell death, represents a paradigm shift in our understanding of vascular pathology. It is increasingly recognized as a hierarchical metabolic disorder integrated within a self-amplifying “bilateral ferroptosis–inflammation circuit”. It has emerged as a critical pathogenic mechanism across a broad spectrum of debilitating vascular diseases, including atherosclerosis, pulmonary hypertension, aortic aneurysm, aortic dissection, and stroke. Our synthesis underscores that ferroptosis bridges vascular aging with acute metabolic collapse, repositioning it as a dynamic pathological dimension rather than an isolated terminal event, and we have detailed how this process drives pathology through a multicellular cascade. The process often begins with the dysfunction of endothelial cells, which initiates the lesion. This is followed by the critical involvement of immune cells, which orchestrate and amplify the pro-ferroptotic environment. Infiltrating macrophages, for instance, create a hostile microenvironment of oxidative stress and iron overload that drives ferroptosis within atherosclerotic plaques and aneurysms. Neutrophils contribute by releasing extracellular traps (NETs) that can directly induce ferroptosis in neighboring smooth muscle cells. Furthermore, the ferroptotic dysfunction of adaptive immune cells, specifically CD4+ T cells in aortic dissection, can cripple the immune response and exacerbate tissue injury. This immune-driven assault often culminates in the death and pathological transformation of vascular smooth muscle cells, which ultimately precipitates the loss of vascular structural integrity. These findings collectively reposition ferroptosis from a peripheral phenomenon to a central driver of disease, firmly establishing its key pathways as highly promising targets for the development of novel, mechanism-based therapeutics.

A key insight emerging from the synthesis of recent research is the remarkable convergence of these diverse vascular pathologies on common cellular and molecular hubs. While immune cells often act as primary instigators, a striking commonality at the execution level is the central role of the vascular smooth muscle cell (VSMC) as a key target. Crucially, we highlight the mitochondria as the essential intermediate hub where bioenergetic failure and lipid remodeling integrate upstream nutrient-sensing dysregulation with downstream membrane rupture [17,131]. This process is governed by metabolic rheostats such as AMPK and SIRT1, whose failure in aged vessels creates a “pro-ferroptotic priming” signal. At the molecular level, this review highlights several shared signaling pathways that represent common vulnerabilities. The most prominent among these pathways is the dysregulation of the canonical SLC7A11/GSH/GPX4 antioxidant axis, which functions as a final common executioner pathway. In contrast, the NRF2 pathway repeatedly appears as a master regulator of the antioxidant response, whose therapeutic activation is a key protective strategy. Upstream of these events, the dysregulation of iron metabolism through mechanisms like NCOA4-mediated ferritinophagy or aberrant TFRC-mediated iron uptake provides the necessary fuel for the ferroptotic cascade. Crucially, inflammatory signaling, such as the damage-associated molecular pattern (DAMP) molecule HMGB1 activating the TLR4 pathway in macrophages during pulmonary hypertension, acts as a critical initiator that directly links sterile inflammation to the execution of ferroptosis. A deeper understanding of these interconnected cellular and molecular events will provide a crucial theoretical basis for developing next-generation therapies targeting these devastating vascular conditions.

Despite the accumulating evidence, there are several limitations. Most of the results are derived from animal or cell models and need to be validated in human studies. The interaction of ferroptosis with other forms of cell death in vascular pathology is unclear, and specialized techniques to detect ferroptosis in human tissues are lacking. Future research should focus on developing specific biomarkers and clinical therapeutics to translate these mechanistic insights into clinical applications.

## Figures and Tables

**Table 1:** Integrated comparison of ferroptosis mechanisms across vascular diseases.

Disease	Target Cells	Antioxidant Defense Axis	Iron Metabolism	Lipid Peroxidation	Immune/Inflammatory Response
AS	Macrophages	(+F) HUA suppresses NRF2/SLC7A11/GPX4 [37] (−F) MCL releases NRF2 from KEAP1/NRF2 complex to increase GPX4 and xCT expression [40] (−F) Tricetin activates NRF2/GPX4 and NRF2/xCT [41] (−F) Melatonin activates NRF2/SLC7A11/GPX4 [42] (−F) MJT activates the SLC7A11/GSH pathway [43]	(−F) MJT decreases DMT1 expression via STAT6 to inhibit iron uptake [43] (−F) MJT increases FTH1 levels to bind free ferrous iron [43]	(+F) ALOX5 and NCF2 upregulation induce ROS generation [35] (+F) Jak2VF erythrophagocytosis delivers lipid hydroperoxides [36] (−F) MJT decreases ACSL4 and LPCAT3 levels [43]	-
	VECs	(+F) PGPC decreases GPX4 and GSH levels via the CD36 receptor [50] (+F) LOX-1 activates cGAS-STING signaling to increase the expression of NCOA4, which suppresses GPX4 and SLC7A11 [53] (−F) Estradiol activates the NRF2/GPX4 pathway [52] (+F) Neu5Ac inhibits the XC-/GSH/GPX4 pathway and promotes SLC3A2 ubiquitination and degradation [54]	(+F) HMOX1 upregulation promotes ferroptosis by releasing free iron [51] (+F) OVX mice show iron accumulation [52]	(−F) The inhibition of ferroptosis by ox-LDL reduces lipid peroxidation [48]	(+F) Ferroptosis increases adhesion molecule expression [48]
	VSMCs	(−F) YAP1 stimulates GLS1 to promote Glu production for GSH synthesis and increases GPX4 activity [59] (−F) Echinatin activates Nrf2 to increase GCLC and GCLM levels for GSH synthesis [60]	-	-	-
PH	PAECs	(+F) NOX4 expression is increased and GPX4 expression is decreased [69] (+F) Erythrophagocytosis decreases GPX4 and SLC7A11 levels [70]	(+F) FTH1 expression is decreased [69]	(+F) Erythrophagocytosis increases lipid peroxidation [70]	PAEC ferroptosis activates the HMGB1/TLR4/NLRP3 pathway [69] (−F) PRDX6 overexpression inhibits HMGB1/TLR4/NLRP3 inflammasome [71]
	Macrophages	-	-	-	(−F) PRDX6 inhibits HMGB1/TLR4/NLRP3 inflammasome and inflammatory cytokine secretion [71]
	PASMCs	(−F) SLC7A11 binds OTUB1 to stabilize itself, increasing GPX4 and GSH levels [74] (−F) circMyst4 combines with DDX5 to promote GPX4 mRNA processing [76] (+F) Circ-calm4 inhibits GPX4 expression [77]	(+F) The circ-calm4/COMP axis increases TFR1 and ferrous iron levels [77]	(−F) circMyst4 suppresses the Eef1a1/ACSL4 interaction [76]	-
AA	Macrophages and Neutrophils	(+F) NETs deplete mitochondrial GSH via SLC25A11 inhibition [84,85]	(+F) Accumulation of labile iron [81,82]	-	
	VSMCs	(−F) Ferrostatin-1 alleviates AAA by activating the SLC7A11/GPX4 axis [92]	(−F) Ganglioside GM3 restricts iron uptake [93] (−F) miR-361-5p adjusts iron-handling proteins [94]	(+) PUFA-PL peroxidation [91]	-
AD	CD4^+^ T cells	SLC7A11/GPX4 axis [95]	-	-	The hypofunctional phenotype of T cells [95]
	VSMCs	(+F) METTL3 upregulation promotes the m6A modification and then the inhibition of SLC7A11 and FSP1 [96,98]	(+F) HIF-1α/HMOX1 releases labile iron [99]	-	-
Stroke	Neurons	(−F) Kaempferol and selenium activates the Nrf2/SLC7A11/GPX4 axis [114,115]	-	(+F) ACSL4 accelerates lipid peroxidation [111]	(+F) I/R triggers the TLR4/NF-κB pathway, causing an ROS burst [110]
	Microglia	-	-	-	(+F) ALPK1 drives ferroptosis via JAK2/STAT3 [125]
	Astrocyte	(+F) NDRG2 upregulation depletes SLC7A11/GSH/GPX4 [121]	(−F) KBA/Z-GS synergistically restore the Fth1 levels [123]	-	Inhibition of the Wnt/β-catenin pathway in the initiation phase [121]
	Oligodendrocytes	The inhibition of the SLC7A11/GSH/GPX4 pathway	-	(+F) PLIN2-mediated lipid remodeling [119] (−F) IL-10 reduces lipid reactive oxygen levels [117]	-
	BMECs	(+F) Nrf2/HO-1 pathway inhibition by HMGB1-mediated LCN2 [129]		-	-
cSVD	BVECs	(+F) VEGF/VEGFR impairment reduces GR and ASS1 activities, causing GSH depletion [119]	-	-	-
	OPC	-	OPCs suffer iron deprivation due to BVEC exhaustion [131]	-	-

(+F) = promotes ferroptosis (pathogenic); (−F) = inhibits ferroptosis (protective/therapeutic).

**Table 2:** Therapeutic agents targeting ferroptosis in cardiovascular and cerebrovascular diseases.

Category	Agent	Role of the Agent	Application	KEY Findings	Model System	Targeted Pathway	Translational Stage	Ref.
Synthetic ferroptosis inhibitors	Fer-1	protective	atherosclerosis	alleviates lesion progression and foam cell formation; reduces the iron content and lipid accumulation via AMPK activation	ox-LDL-treated macrophages/foam cells	AMPK	preclinical (in vitro)	[134]
		protective	stroke	reduces iron accumulation; prevents neuronal loss; improves neurological outcomes; crosses the blood–brain barrier; inhibits lipid peroxidation	mouse model of cerebral ischemia/reperfusion injury	ACSL4	preclinical (in vitro)	[135]
		protective	aortic aneurysm/dissection	preserves aortic wall integrity via MEF2C/KDM5A modulation; alters miRNA expression	aortic dissection mouse model	MEF2C/KDM5A	preclinical (in vitro)	[94]
	Lipro-1	protective	intracerebral hemorrhage	GPX4 restoration during ferroptosis; potential therapeutic application	rat model of intracerebral hemorrhage	GPX4	preclinical (in vitro)	[136]
Iron chelation therapy	DFO	protective	ischemic stroke	intranasal administration reduces infarct volume; chelates free iron; attenuates lipid peroxidation	rat model of ischemic stroke	iron chelation (Fenton reaction)	preclinical (in vitro)	[137]
		protective	intracerebral hematoma	accelerates recovery; reduces hematoma volume	human patients (meta-analysis)	iron chelation	clinical (meta-analysis)	[138]
Natural products	QCT	protective	postmenopausal atherosclerosis	activates NRF2/GPX4 signaling via KEAP1 ubiquitination; inhibits endothelial cell ferroptosis	ovariectomized mouse models of atherosclerosis and endothelial cells	KEAP1/NRF2/GPX4	preclinical (in vivo & in vitro)	[139]
	SAL-A	protective	ischemic stroke	inhibits ferroptosis via Nrf2-related pathways	ischemic stroke animal model	Nrf2	preclinical (in vivo)	[140]
		protective	intracerebral hemorrhage	inhibits ferroptosis via the Akt/GSK-3β/Nrf2 signaling pathway	intracerebral hemorrhage animal model	Akt/GSK-3β/Nrf2	preclinical (in vivo)	[141]

## Data Availability

No new data were created or analyzed in this study. Data sharing is not applicable to this article.

## Abbreviations

The following abbreviations are used in this review:

4-HNE	4-Hydroxynonenal
AA	Aortic aneurysm
AAA	Abdominal aortic aneurysm
ACSL4	Acyl-CoA synthetase long-chain family member 4
AD	Aortic dissection
ALOX5	5-Lipoxygenase
ALOXs	Lipoxygenases
ALPK1	Alpha-kinase 1
AS	Atherosclerosis
BBB	Blood–brain barrier
BVECs	Brain vessel endothelial cells
CoQ10	Ubiquinone
CoQ10H_2_	Ubiquinol
CSE	Cigarette smoke extract
cSVD	Cerebral small vessel disease
DAMPs	Damage-associated molecular patterns
DDX5	DEAD box helicase 5
DFO	Deferoxamine
DHFR	Dihydrofolate reductase
DMT1	Ferrous ion membrane transport protein DMT1
E2	Estradiol
Eef1a1	Eukaryotic translation elongation factor 1 alpha 1
EP	Erythrophagocytosis
Fer-1	Ferrostatin-1
FPN	Ferroportin
FSP1	Ferroptosis suppressor protein 1
FTH1	Ferritin heavy chain 1
GCLC	Catalytic subunit of glutamate cysteine ligase
GCLM	Modulatory subunit of glutamate cysteine ligase
GLS1	Glutaminase 1
Glu	Glutamate
GPX4	Glutathione peroxidase 4
GSH	Glutathione
HA	Hyaluronic acid
HFD	High-fat diet
HHcy	Hyperhomocysteinemia
HIF-1α	Hypoxia-inducible factor-1α
HMGB1	High-mobility group box 1
HPH	Hypoxic pulmonary hypertension
HUA	High levels of uric acid
HUVECs	Human umbilical vein endothelial cells
I/R	Ischemia–reperfusion
IL-10	Interleukin-10
KBA	11-Keto-β-boswellic acid
LIP	Labile iron pool
Lipro-1	Liproxstatin-1
LPCAT3	Lysophosphatidylcholine acyltransferase 3
MAECs	Mouse aortic endothelial cells
MALT1	Mucosa-associated lymphoid tissue lymphoma translocation protein 1
MCL	Micheliolide
MCT	Monocrotaline
MDA	Malondialdehyde
METTL3	Methyltransferase-like 3
MJT	Maijitong granule
MSC-EVs	Mesenchymal stem cell-derived extracellular vesicles
NADPH	Nicotinamide adenine dinucleotide phosphate
NCOA4	Nuclear receptor coactivator 4
NCF2	P67phox
NDRG2	N-myc downstream-regulated gene 2
NETs	Neutrophil extracellular traps
Neu5Ac	N-Acetylneuraminic acid
NLRP3	NOD-like receptor family pyrin domain containing 3
NOXs	NADPH oxidase
NPs	Nanoparticles
OGD/R	Oxygen–glucose deprivation/reoxygenation
OPCs	Oligodendrocyte progenitor cells
OTUB1	Ubiquitin aldehyde binding 1
OVX	Ovariectomized
PAECs	Pulmonary artery endothelial cells
PASMCs	Pulmonary artery smooth muscle cells
PGPC	1-Palmitoyl-2-glutaroyl-sn-glycero-3-phosphocholine
PH	Pulmonary hypertension
PLGA	Poly(lactic-co-glycolic) acid
PLIN2	Perilipin-2
PLOOH	Lipid peroxides
PMVECs	Pulmonary microvascular endothelial cells
PUFA-PLs	Polyunsaturated fatty acid-containing phospholipids
PUFAs	Polyunsaturated fatty acids
QCT	Quercetin
ROS	Reactive oxygen species
RvD1	Resolvin D1
SAL-A	Salvianolic acid A
SCARB1	Scavenger receptor class B member 1
SCI	Spinal cord injury
SE	Superenhancer
SESN1	Sestrin 1
TfR	Transferrin receptor
TfR1	Transferrin receptor 1
TLR4	Toll-like receptor 4
TRI	Tricetin
TRPML 1	Mucolipin 1
VCI	Vascular cognitive impairment
VECs	Vascular endothelial cells
VSMCs	Vascular smooth muscle cells
WMI	White matter injury
xCT	Xap5 circadian timekeeper
YAP1	Yes-associated protein 1
Z-GS	Z-Guggulsterone
